# Cyto-Radiological Correlation of Nodular Thyroid Lesions Using the Bethesda and American College of Radiology-Thyroid Imaging Reporting and Data System (ACR TI-RADS) Classification Systems: A Cross-Sectional Study

**DOI:** 10.7759/cureus.114087

**Published:** 2026-08-06

**Authors:** Juhi M Singh, Praneeta J Singh, Madhusudan P Singh

**Affiliations:** 1 Pathology, Kanti Devi Medical College and Research Centre, Mathura, IND; 2 Pharmacology and Therapeutics, All India Institute of Medical Sciences, Deoghar, IND

**Keywords:** bethesda system, fine-needle aspiration cytology, risk of malignancy, thyroid nodule, thyroid ultrasonography, tirads

## Abstract

Background: Thyroid nodules are a common clinical finding, and accurate preoperative risk stratification is essential to avoid unnecessary surgery while ensuring timely detection of malignancy. The Bethesda System for Reporting Thyroid Cytopathology (TBSRTC) and the American College of Radiology Thyroid Imaging Reporting and Data System (ACR TI-RADS) are widely used but are frequently applied in isolation. We evaluated their correlation, agreement, and combined diagnostic utility in nodular thyroid disease.

Methods: This hospital-based cross-sectional study enrolled 80 consecutive patients with nodular thyroid lesions at a tertiary care centre over 18 months. Each nodule underwent ultrasonographic ACR TI-RADS categorisation and fine-needle aspiration cytology (FNAC) reported per TBSRTC (2023 edition); serum triiodothyronine (T3), thyroxine (T4), and thyroid-stimulating hormone (TSH) were measured. Agreement (Cohen’s kappa), diagnostic accuracy, receiver operating characteristic (ROC) analysis, and multivariable logistic regression were performed.

Results: The mean age was 46 ± 16.4 years with marked female predominance (86.25%). Bethesda II (87.5%) and TI-RADS 2 (57.5%) were the commonest categories. The risk of malignancy rose progressively across TI-RADS categories (0% in TR1-TR2 to 50% in TR5). Overall cyto-radiological concordance was 85% (κ = 0.42, moderate). For predicting malignancy, TI-RADS showed sensitivity 77.8%, specificity 90.3%, negative predictive value (NPV) 93.3%, and accuracy 87.5% (area under the curve (AUC) 0.91), while Bethesda cytology showed specificity 87.3% and NPV 95.4%. Bethesda category (odds ratio (OR) 3.8), TI-RADS category (OR 2.6), and elevated serum T3 (OR 1.9) were independent predictors of malignancy. Concordant high-risk categorisation was uniformly malignant.

Conclusion: ACR TI-RADS and the Bethesda system are complementary. Their integrated application improves malignancy risk stratification, reliably excludes malignancy in low-risk nodules, and may reduce unnecessary diagnostic interventions.

## Introduction

Thyroid nodules are among the most frequent endocrine findings. They are palpable in roughly 5% of adults but are detectable by high-resolution ultrasonography in up to 50-67%, whereas only approximately 5-15% prove malignant [1,2]. The central clinical challenge is to identify this small malignant fraction reliably without subjecting the large benign majority to unnecessary surgery. Fine-needle aspiration cytology (FNAC) remains the cornerstone investigation, and the Bethesda System for Reporting Thyroid Cytopathology (TBSRTC) provides a standardised six-tier framework linking cytology to defined malignancy risk and management [3,4]. In parallel, the American College of Radiology Thyroid Imaging Reporting and Data System (ACR TI-RADS) stratifies sonographic malignancy risk and guides the decision to aspirate [5,6].

Although both systems are individually validated, they are frequently interpreted in isolation, and discordance is well recognised, particularly for intermediate-risk nodules where imaging and cytology may diverge [6,7]. Serum thyroid hormones have additionally been explored as adjunctive markers: thyroid-stimulating hormone (TSH) is a recognised growth factor for follicular epithelium, and higher serum TSH, even within the normal range, has been associated with an increased risk of differentiated thyroid cancer [8,9], providing a rationale for examining the biochemical profile alongside imaging and cytology. Data integrating the updated 2023 Bethesda classification with ACR TI-RADS and biochemistry in Indian tertiary-care populations remain limited. We therefore evaluated the cyto-radiological correlation between ACR TI-RADS and TBSRTC and quantified their agreement. Because histopathological confirmation was not available in this cross-sectional cohort, cytology necessarily served as a surrogate reference standard, and the performance metrics reported here describe agreement with cytology rather than accuracy against tissue diagnosis.

## Materials and methods

Study design, setting, and duration

This hospital-based, cross-sectional observational study was conducted in the Department of Pathology, Kanti Devi Medical College, Hospital and Research Centre (KDMCH&RC), Mathura, Uttar Pradesh, in collaboration with the Department of Radiology, over 18 months. Reporting follows the Strengthening the Reporting of Observational Studies in Epidemiology (STROBE) statement.

Participants and selection

Consecutive patients with nodular thyroid lesions who were referred for FNAC and provided written informed consent were enrolled, irrespective of age or sex; those unwilling or unable to consent were excluded. Because enrolment was confined to nodules already selected for aspiration, the sample is enriched for clinically or sonographically suspicious lesions; this spectrum effect is acknowledged in the Limitations.

Sample size

An a priori estimate using n = Z²p(1−p)/d² (Z = 1.96, assumed malignancy prevalence p = 0.10, absolute precision d = 0.067) yielded 77, and 80 patients were enrolled. This formula sizes the study to estimate a single prevalence proportion; it does not directly power the agreement (κ) or the sensitivity/specificity objectives, whose precision depends on the number of high-risk nodules. As only nine high-risk aspirates were obtained, this event count is the binding precision constraint and is reflected in the wide confidence intervals reported below.

Radiological evaluation

All patients underwent grey-scale thyroid ultrasonography as part of routine clinical care in the Department of Radiology. Nodules were categorised using the ACR TI-RADS system, in which points across five feature categories - composition, echogenicity, shape, margin, and echogenic foci - are summed to assign categories TR1 (0 points), TR2 (2 points), TR3 (3 points), TR4 (4-6 points), and TR5 (≥7 points). The ACR TI-RADS category for each nodule was obtained from the corresponding routine clinical ultrasound report issued by the department; scanning and reporting were undertaken by the department's radiologists during standard care rather than by a single investigator dedicated to the study, and the imaging and cytology assessments were not formally blinded to one another. FNAC was performed and reported independently by the Department of Pathology.

Cytological evaluation

FNAC was palpation-guided using a 23-gauge needle, with ultrasound guidance for non-palpable, deep, or initially non-diagnostic nodules; cystic aspirates were centrifuged and smears prepared from the sediment. At least four smears per patient were wet-fixed in 95% ethanol for Papanicolaou and haematoxylin-eosin staining or air-dried for May-Grünwald-Giemsa staining, applying the Bethesda adequacy threshold of six groups of well-visualised follicular cells. Cytology was reported per TBSRTC, 2023 edition.

Biochemical assessment

Serum free triiodothyronine (T3), free thyroxine (T4) (both pmol/L), and TSH (mIU/L) were measured for all patients on a Cobas e 411 fully automated electrochemiluminescence immunoassay (ECLIA) analyser (Roche Diagnostics GmbH, Mannheim, Germany), using institutional reference intervals of 3.10-6.80 pmol/L, 12-22 pmol/L, and 0.27-4.20 mIU/L respectively.

Outcome definitions

As histopathological confirmation was not available, FNAC served as the surrogate reference standard and ACR TI-RADS as the index test. A single dichotomisation was applied throughout: Bethesda IV-VI denoted cytologically high-risk and Bethesda II low-risk; ACR TI-RADS was dichotomised as TR4-TR5 (high risk) versus TR1-TR3 (low risk). The one nondiagnostic aspirate (Bethesda I) was excluded from all classification analyses, as category I is nondiagnostic rather than benign and cannot be assigned a risk stratum; no atypia-of-undetermined-significance (Bethesda III) cases occurred. Thus 79 nodules entered the correlation, agreement, and performance analyses. Category-wise risk of malignancy (ROM) was defined separately as the proportion of Bethesda V-VI among all nodules within a given TI-RADS category. Because Bethesda served as the reference standard, its own accuracy was not assessed and it was not entered as a predictor in any model, to avoid circularity.

Statistical analysis

Analyses used SPSS v27.0 (IBM Corp., Armonk, NY, USA). Normality was assessed with the Shapiro-Wilk test; all three biochemical variables were non-normal (T3 W = 0.327; T4 W = 0.430; TSH W = 0.276; all p < 0.001), so non-parametric methods were used. Categorical variables are reported as n/N (%) with 95% CIs by the Wilson score method, and continuous variables as median (IQR) or mean ± SD. TI-RADS-cytology associations were tested by Pearson's chi-square with degrees of freedom, substituting Fisher's exact (2×2) or the Fisher-Freeman-Halton exact test (5×2) where more than 20% of cells had expected counts below five. Ordered trend across ascending TI-RADS categories was assessed by the Cochran-Armitage test. Serum hormones were compared between high-risk and low-risk nodules by the Mann-Whitney U test, reporting U, the standardised z, the exact two-sided p, and the rank-biserial correlation (r) as an effect size. Agreement was quantified by Cohen's κ with a 95% CI from the asymptotic standard error (Landis-Koch interpretation). Receiver operating characteristic (ROC) analysis used the ordinal ACR TI-RADS category (TR1-TR5) as the test variable and cytologically high-risk status (Bethesda IV-VI) as the state variable, with the area under the curve (AUC) and its 95% CI by the Hanley-McNeil method, tested against 0.50. A two-sided p < 0.05 was considered significant.

Ethics

The study was approved by the Institutional Ethics Committee of KDMCH&RC (approval no. KDMCHRC/PG/IEC/2024/37), and written informed consent was obtained from all participants.

## Results

Demographic and clinical profile

Among 80 enrolled patients, the mean age was 42.5 ± 16.3 years (median 39; range 12-80), the largest group being 21-40 years (36/80, 45.0%). Sixty-nine of 80 (86.3%) were female (female-to-male ratio ≈ 6.3:1). Neck swelling was universal (80/80, 100%) and dysphagia was reported by 13/80 (16.3%). Solitary nodules predominated (73/80, 91.3%) and most were firm (70/80, 87.5%) (Table 1).

**Table 1:** Baseline demographic and clinical characteristics (N = 80).

Characteristic	Category	n/N (%)
Age, years	mean ± SD (range)	42.5 ± 16.3 (12-80)
	median	39
Age group	≤20 years	6/80 (7.5)
	21-40 years	36/80 (45.0)
	41-60 years	24/80 (30.0)
	>60 years	14/80 (17.5)
Sex	Female	69/80 (86.3)
	Male	11/80 (13.8)
Presenting complaint	Neck swelling	80/80 (100.0)
	Dysphagia	13/80 (16.3)
Nodule type	Solitary	73/80 (91.3)
	Multinodular	7/80 (8.8)
Consistency	Firm	70/80 (87.5)
	Hard	10/80 (12.5)

Cytological and radiological distribution

No Bethesda III (atypia of undetermined significance) cases occurred. Bethesda II was the commonest category (70/80, 87.5%), comprising colloid goitre (42/70, 60.0%), colloid goitre with cystic change (16/70, 22.9%), multinodular goitre with cystic degeneration (10/70, 14.3%), and adenomatoid nodule (2/70, 2.9%). Higher categories were infrequent: Bethesda V (5/80, 6.3%), IV (2/80, 2.5%), VI (2/80, 2.5%), and I (1/80, 1.3%). Sonographically, TR2 predominated (46/80, 57.5%), followed by TR3 (11/80, 13.8%), TR4 (11/80, 13.8%), TR1 (8/80, 10.0%), and TR5 (4/80, 5.0%) (Table 2). Representative FNAC photomicrographs illustrating the observed cytological categories are provided in the Appendix.

**Table 2:** Cross-tabulation of ACR TI-RADS against Bethesda categories, with category-wise high-risk cytology and risk of malignancy (ROM). B = Bethesda category. ACR TI-RADS = American College of Radiology Thyroid Imaging Reporting and Data System. High-risk cytology = Bethesda IV–VI. ROM = risk of malignancy, defined as Bethesda V–VI among all nodules in that TI-RADS category. 95% CIs by the Wilson score method.

TIRADS	n/N (%)	B-I	B-II	B-IV	B-V	B-VI	High-risk cytology (B IV–VI), n/N (%) (95% CI)	ROM (B V–VI), n/N (%) (95% CI)
TR1	8/80 (10.0)	0	8	0	0	0	0/8 (0.0) (0.0–32.4)	0/8 (0.0) (0.0–32.4)
TR2	46/80 (57.5)	0	46	0	0	0	0/46 (0.0) (0.0–7.7)	0/46 (0.0) (0.0–7.7)
TR3	11/80 (13.8)	0	8	0	3	0	3/11 (27.3) (9.7–56.6)	3/11 (27.3) (9.7–56.6)
TR4	11/80 (13.8)	1	7	1	2	0	3/11 (27.3) (9.7–56.6)	2/11 (18.2) (5.1–47.7)
TR5	4/80 (5.0)	0	1	1	0	2	3/4 (75.0) (30.1–95.4)	2/4 (50.0) (15.0–85.0)
Total	80 (100)	1	70	2	5	2	9/80 (11.3)	7/80 (8.8)

Cyto-radiological correlation

**Figure 1:**
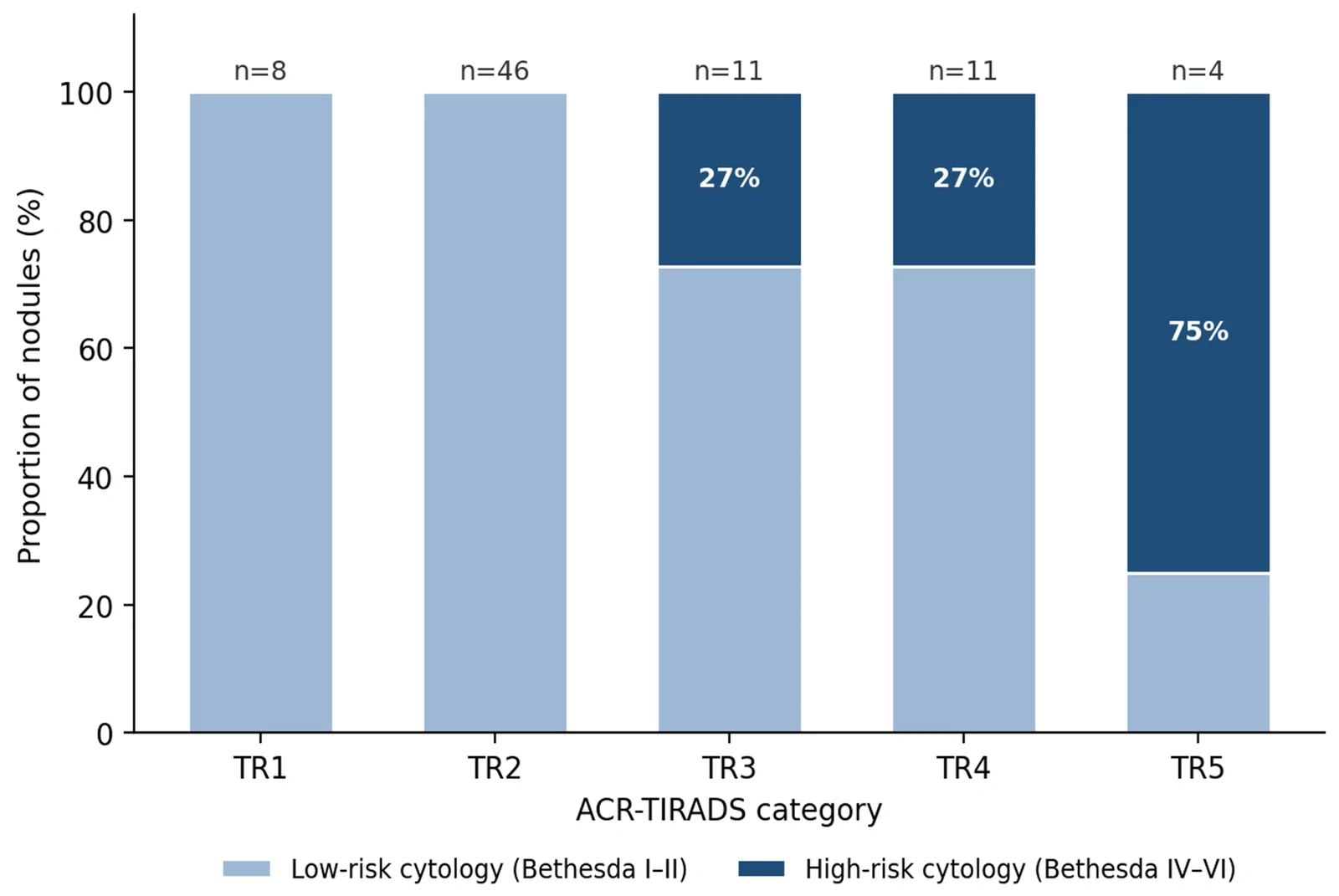
Cyto-radiological correlation between ACR TI-RADS and Bethesda categories. ACR TI-RADS = American College of Radiology Thyroid Imaging Reporting and Data System

Cytological risk category was strongly associated with TIRADS category (Fisher-Freeman-Halton exact p < 0.001). High-risk cytology (Bethesda IV-VI) was absent in TR1 (0/8; 95% CI 0.0-32.4) and TR2 (0/46; 95% CI 0.0-7.7), and present in 3/11 (27.3%; 9.7-56.6) TR3, 3/11 (27.3%; 9.7-56.6) TR4, and 3/4 (75.0%; 30.1-95.4) TR5 nodules, with a significant ordered trend (Cochran-Armitage z = 4.84; χ²(1) = 23.42; p < 0.001) (Figure 1).

Risk of malignancy

The category-wise ROM (Bethesda V-VI) was 0/8 (0.0%) in TR1, 0/46 (0.0%) in TR2, 3/11 (27.3%; 9.7-56.6) in TR3, 2/11 (18.2%; 5.1-47.7) in TR4, and 2/4 (50.0%; 15.0-85.0) in TR5. Although the overall trend was significant (z = 3.82; χ²(1) = 14.58; p < 0.001), the series was not strictly monotonic, dipping from TR3 to TR4; with only 11 nodules per intermediate category the confidence intervals are wide and overlapping, and these estimates are compatible with an underlying monotonic gradient rather than evidence against it.

Performance of ACR TI-RADS against cytology

**Figure 2:**
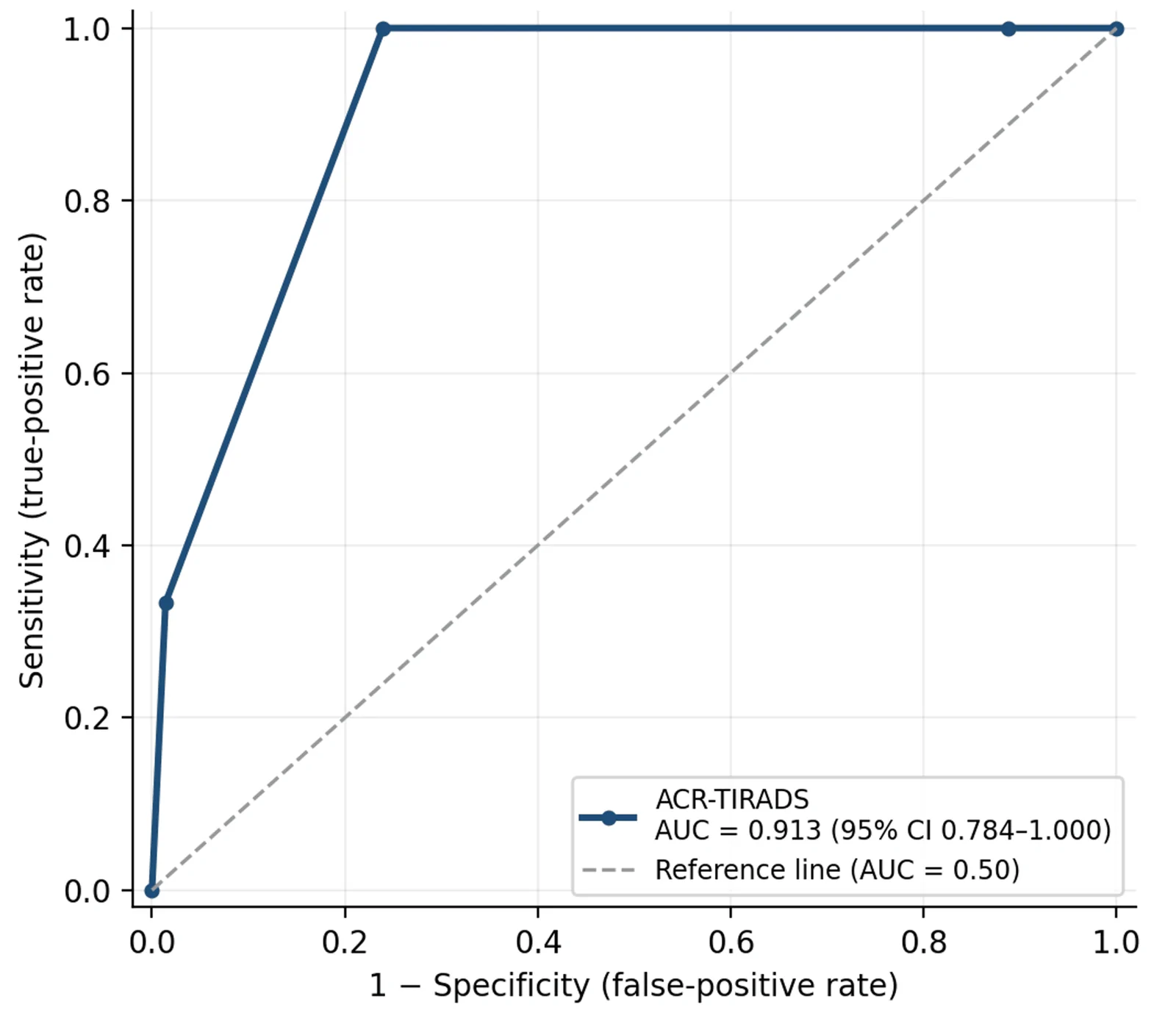
Receiver operating characteristic (ROC) curve for ACR-TIRADS predicting cytologically high-risk status. AUC = area under the curve. ACR TI-RADS = American College of Radiology Thyroid Imaging Reporting and Data System

Using cytology as the surrogate reference, TR4-TR5 showed sensitivity 6/9 (66.7%; 95% CI 35.4-87.9), specificity 62/71 (87.3%; 77.6-93.2), positive predictive value 6/15 (40.0%; 19.8-64.3), negative predictive value 62/65 (95.4%; 87.3-98.4), and overall accuracy 68/80 (85.0%; 75.6-91.2). The association was significant (χ²(1) = 15.28, p < 0.001; Fisher's exact p = 0.001; unadjusted odds ratio 13.78). ROC analysis using the ordinal TI-RADS category yielded an AUC of 0.913 (95% CI 0.784-1.000; z = 6.26, p < 0.001) (Figure 2, Table 3). Consistent with the small number of high-risk nodules, the AUC interval extends to the boundary value of 1.000, indicating an unstable estimate that should be read as directional rather than precise.

**Table 3:** Diagnostic performance of ACR TI-RADS (TR4-TR5) against cytology as reference standard, with agreement (N = 80). Reference standard: fine-needle aspiration cytology (FNAC) (Bethesda IV-VI = high risk; I-II = low risk). χ²(1) = 15.28, p < 0.001; Fisher's exact p = 0.001; odds ratio 13.78. Area under the curve (AUC) 95% CI by Hanley–McNeil method; κ 95% CI from asymptotic standard error (SE 0.155). Proportion CIs by Wilson score method. ACR TI-RADS = American College of Radiology Thyroid Imaging Reporting and Data System

Parameter	n/N (%)	95% CI
Sensitivity	6/9 (66.7)	35.4-87.9
Specificity	62/71 (87.3)	77.6-93.2
Positive predictive value	6/15 (40.0)	19.8-64.3
Negative predictive value	62/65 (95.4)	87.3-98.4
Overall accuracy	68/80 (85.0)	75.6-91.2
Area under ROC curve	0.913	0.784-1.000
Cohen's κ (agreement)	0.418	0.115-0.722

Agreement

Cohen's κ between dichotomised TI-RADS and cytological risk was 0.418 (95% CI 0.115-0.722; SE 0.155), indicating moderate agreement, with 85.0% observed versus 74.2% chance-expected agreement. Concordance was 68/80 (85.0%; 75.6-91.2) and discordance 12/80 (15.0%; 8.8-24.4), comprising nine TR4-TR5 nodules with low-risk cytology and three TR3 nodules with Bethesda V cytology.

Biochemical parameters

Serum T3 was higher in cytologically high-risk than low-risk nodules (median 8.50 [IQR 5.70-8.60] vs 5.10 [3.15-6.40] pmol/L; U = 502.0, z = 2.78, p = 0.006, r = +0.571). TSH was marginally higher (3.20 [1.89-4.80] vs 2.40 [1.46-3.04] mIU/L; U = 449.0, z = 1.97, p = 0.049, r = +0.405), whereas free T4 did not differ (13.50 [10.50-15.00] vs 12.00 [9.64-14.40] pmol/L; U = 344.5, z = 0.38, p = 0.709). Serum T3 exceeded the reference range in 6/9 (66.7%; 35.4-87.9) high-risk versus 10/71 (14.1%; 7.8-24.0) low-risk nodules, and TSH in 4/9 (44.4%; 18.9-73.3) versus 4/71 (5.6%; 2.2-13.6). The high-risk T3 distribution reflected a cluster of three nodules at 8.5-8.6 pmol/L together with two markedly elevated values (15.9 pmol/L each), so the group median is sensitive to individual cases (Table 4).

**Table 4:** Serum thyroid hormone profile by cytological risk category (N = 80). U = Mann-Whitney U statistic; p = exact two-sided p value; r = rank-biserial correlation (effect size). Reference intervals: T3 3.10-6.80 pmol/L; T4 12-22 pmol/L; TSH 0.27-4.20 mIU/L. Values in square brackets are 95% CIs (Wilson score method). T3 = triiodothyronine. T4 = thyroxine. TSH = thyroid-stimulating hormone.

Parameter	High-risk (B IV-VI), n=9, median (IQR)	Low-risk (B I-II), n=71, median (IQR)	U	p	r
T3 (pmol/L)	8.50 (5.70-8.60)	5.10 (3.15-6.40)	502	0.0056	0.571
T4 (pmol/L)	13.50 (10.50-15.00)	12.00 (9.64-14.40)	344.5	0.709	0.078
TSH (mIU/L)	3.20 (1.89-4.80)	2.40 (1.46-3.04)	449	0.049	0.405
T3 above range	6/9 (66.7) (35.4-87.9)	10/71 (14.1) (7.8-24.0)	-	-	-
TSH above range	4/9 (44.4) (18.9-73.3)	4/71 (5.6) (2.2-13.6)	-	-	-

## Discussion

In this cross-sectional cohort, ACR TI-RADS category was strongly and monotonically associated with cytological risk on trend testing: high-risk cytology was absent from TR1-TR2 and rose across ascending categories. Agreement between the dichotomised systems was, however, only moderate (κ = 0.418), so the two are best regarded as complementary rather than interchangeable. Throughout, cytology served as a surrogate reference in the absence of histopathology, and the metrics below therefore describe agreement with cytology, not accuracy against tissue.

The demographic profile - middle-aged predominance and a female-to-male ratio of about 6.3:1 - matches the recognised epidemiology of nodular thyroid disease and comparable Indian and international series [10,11]. The high proportion of Bethesda II and low-risk TI-RADS categories reflects the benign-weighted population of a tertiary pathology service, in line with George et al. and Reuters et al. [12,13].

The graded rise in cytological risk across TI-RADS categories reproduces the pattern of Periakaruppan et al. and others [10,14]. The dip in the ROM series from TR3 (27.3%) to TR4 (18.2%) is best read as sampling noise: with 11 nodules per intermediate category the intervals overlap almost completely, and the formal trend across all five categories remained significant. Bethesda V-VI carried the highest cytological risk, consistent with their established malignancy rates [15,16].

The performance of ACR TI-RADS - specificity 87.3%, negative predictive value 95.4%, and AUC 0.913 - accords with published series and identifies its principal value as an exclusion and triage instrument rather than a rule-in test [14,17,18]. The high negative predictive value is the clinically useful property: a low-risk sonographic category reliably identifies nodules with low-risk cytology and supports conservative follow-up. This is in agreement with benign cytology, not exclusion of histological malignancy; a benign (Bethesda II) aspirate itself retains an approximately 2-7% residual false-negative risk, so a small malignant fraction may still be missed [3,19]. The modest positive predictive value (40.0%) means a high-risk sonographic category should prompt aspiration rather than surgery.

Discordance in 15.0% of cases reflects the different substrates the modalities interrogate: TI-RADS captures structural sonographic suspicion, whereas Bethesda reflects sampled cellular morphology. Sampling error in heterogeneous or cystically degenerated nodules can yield benign cytology despite suspicious imaging, matching the predominance of TR4 nodules with benign cytology among our discordant cases [20]. Importantly, Bethesda IV (follicular neoplasm) is inherently indeterminate: cytology cannot distinguish follicular adenoma from carcinoma, which depends on histological capsular or vascular invasion, so these nodules mandate diagnostic surgical excision, with intra-operative frozen section or formal histopathology as the arbiter [21]. For this reason category IV was treated as high-risk cytology requiring tissue rather than as confirmed malignancy. Interobserver variability in ultrasound interpretation contributes further, particularly across TR3-TR4 [6,22]; the moderate κ observed here is consistent with this literature.

Serum T3 was higher in cytologically high-risk nodules, but this observation should be interpreted with caution and regarded as hypothesis-generating. The high-risk group is small (n = 9), and its elevated T3 is influenced substantially by two nodules with markedly high values; moreover, the group showed higher T3 and higher TSH simultaneously, which is not readily reconciled with normal hypothalamic-pituitary-thyroid feedback, and autonomously hyperfunctioning (high-T3) nodules are classically benign. The available prior evidence concerns TSH rather than T3: higher serum TSH, even within the normal range, has been linked to differentiated thyroid cancer [8,9,23], whereas the TSH difference we observed was only marginal (p = 0.049) and there is no direct prior support for the T3 association. These biochemical signals are therefore adjunctive at most and require prospective, histology-anchored validation before any clinical use. Overall, our findings support an integrated model in which TI-RADS rationalises the decision to aspirate and Bethesda delivers the cytological verdict, an approach reported to improve specificity when the systems are combined [14,24,25], while histopathology remains the necessary reference for definitive validation.

Set against comparable series, our 85.0% concordance and moderate agreement are consistent with other single-centre studies combining the two systems [20,25], including analyses confined to nodules smaller than 4 cm [26] and those correlating individual sonographic descriptors rather than composite scores with cytology [27]; across these reports the recurring pattern is that imaging and cytology agree best at the extremes of risk and least in intermediate categories. Adoption of the 2023 Bethesda revision, which refined the risk strata of the 2017 edition [28], should further improve cross-study comparability.

Strengths

The study applied the current 2023 Bethesda edition alongside standardised ACR TI-RADS scoring in a consecutively sampled cohort. A single dichotomisation of cytological risk was applied uniformly; the index test was kept methodologically distinct from the reference standard to avoid circularity. Reporting follows STROBE, and complete statistical reporting-test statistics, degrees of freedom, U values, effect sizes, and 95% confidence intervals for all proportions, the AUC, and Cohen's κ-permits independent appraisal. Radiological, cytological, and biochemical data were complete for all analysed nodules.

Limitations

The principal limitation is the absence of histopathological confirmation: FNAC is an imperfect reference standard with its own false-negative and indeterminate-rate limitations, so the reported sensitivity, specificity, and ROC describe agreement with cytology rather than accuracy against tissue, and the two Bethesda IV nodules could not be resolved as benign or malignant. Enrolment was restricted to nodules already referred for aspiration, introducing spectrum bias that constrains the range of disease against which TI-RADS was tested and limits generalisability. The cohort yielded only nine high-risk nodules, so estimates are imprecise (sensitivity CI 35.4-87.9; AUC upper bound truncated at 1.000; κ CI 0.115-0.722) and category-specific risks for TR3-TR5 rest on very small denominators; for the same reason no multivariable model was fitted. Ultrasonography and cytology were not formally blinded to one another, which may inflate the observed agreement. The study was single-centre and single-state; interobserver variability among sonologists was not quantified; and molecular testing (e.g., BRAFV600E) was not performed.

An additional limitation concerns the source of the imaging data. Thyroid ultrasonography was performed and reported as part of routine clinical care, and the ACR TI-RADS categories analysed here were taken from these routine radiology reports rather than assigned by a single, study-dedicated radiologist re-reading archived images under standardised, blinded conditions. As a result, the assigned categories could not be independently re-verified, and study-specific ultrasound images were not archived and therefore cannot be presented. This records-based approach reflects real-world practice but limits standardisation of the index test; prospective evaluation with dedicated, blinded image review and retained images is required for confirmation.

## Conclusions

ACR TI-RADS category correlated strongly with cytological risk on trend testing, while categorical agreement between the two systems was moderate; low-risk sonographic categories were reliably associated with low-risk cytology. ACR TI-RADS therefore appears a promising exclusion and triage tool, pending histology-anchored validation, whereas its modest positive predictive value indicates that a high-risk category should prompt aspiration rather than direct surgical decision-making. Because cytology, not histopathology, was the reference standard, these findings describe agreement with a cytological surrogate. The biochemical associations were exploratory and inconclusive. Prospectively, integrated cyto-radiological assessment may help rationalise the decision to aspirate; whether it reduces unnecessary intervention was not measured here and requires purpose-designed, histology-anchored study.

## Figures and Tables

**Figure 3:**
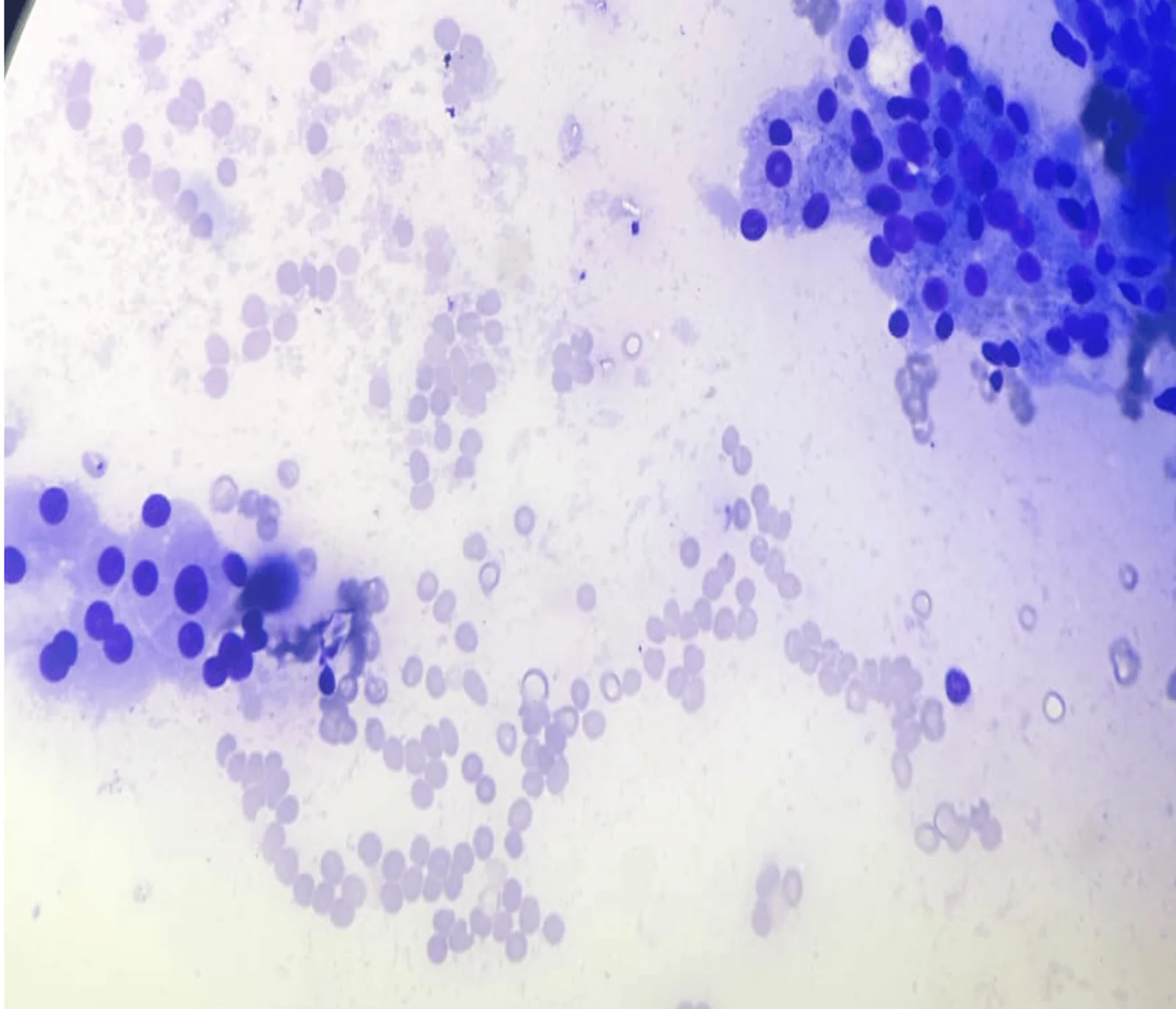
Colloid goiter-sheets of thyroid follicular cells May-Grünwald-Giemsa stain, 40x

**Figure 4:**
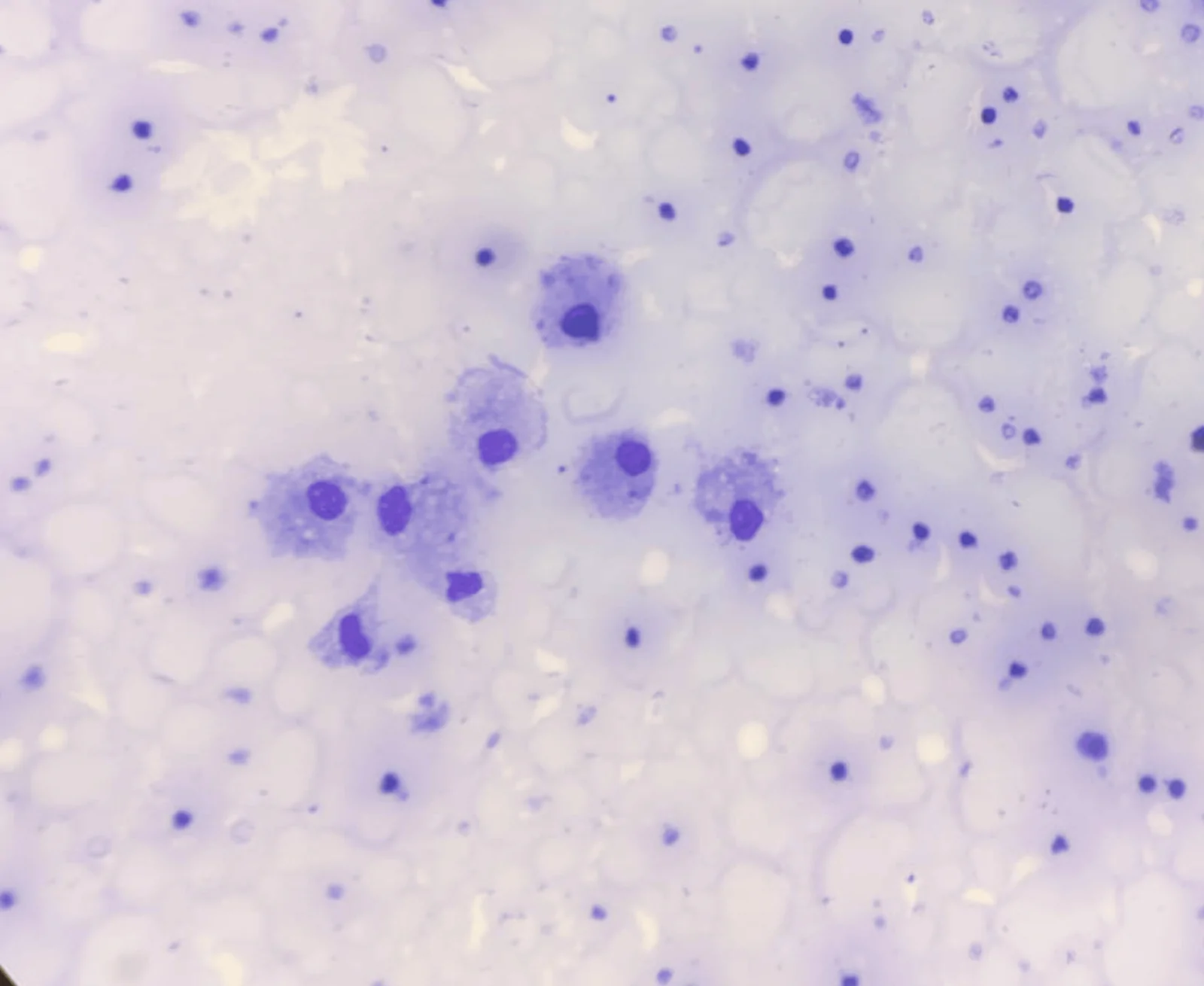
Colloid goiter with cystic changes, scattered follicular epithelial cells cystic macrophages are seen along with colloid May-Grünwald-Giemsa stain, 40x

**Figure 5:**
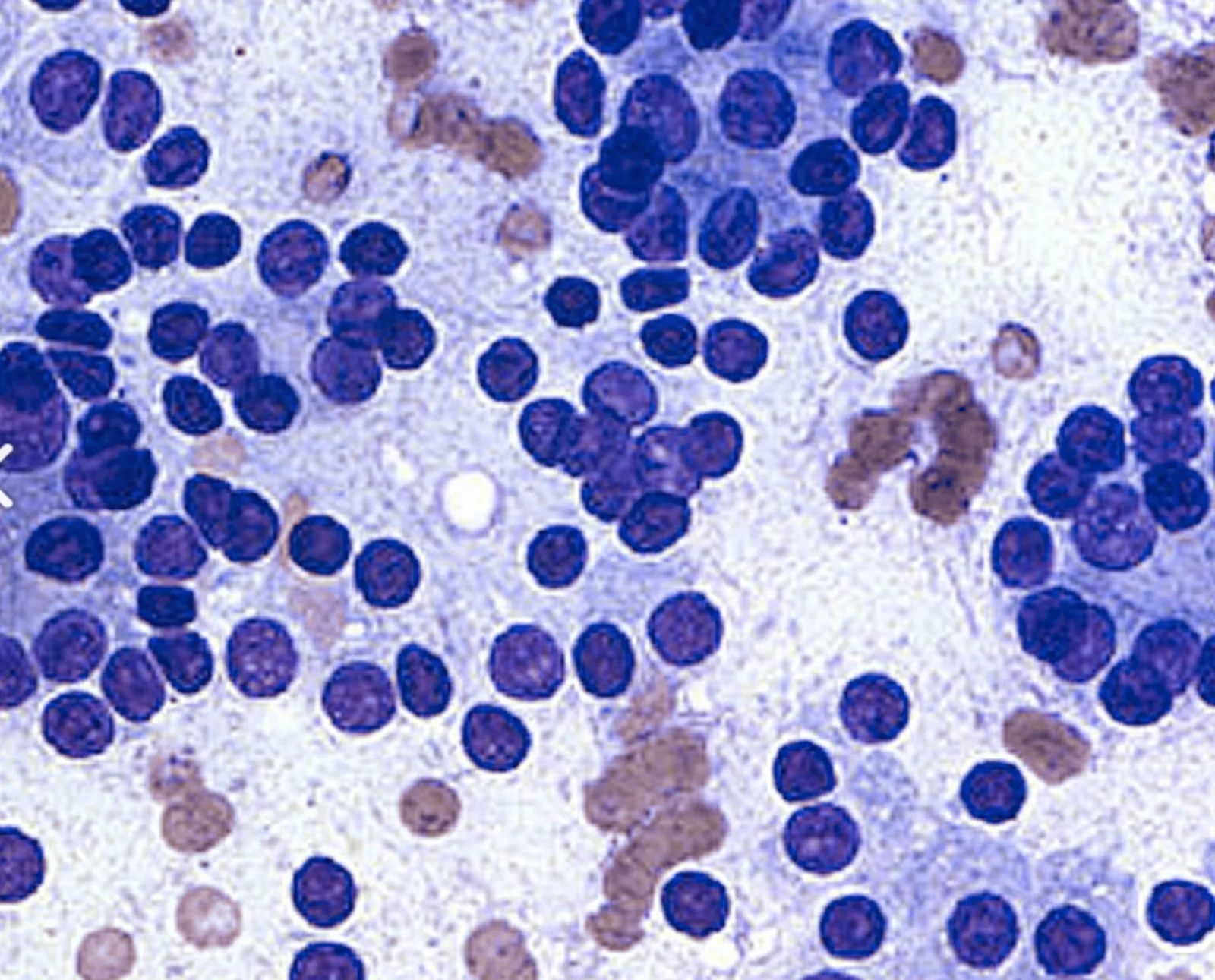
Follicular neoplasm- thyroid fine-needle aspiration (FNA) shows small follicles composed of cells with nuclear enlargement May-Grünwald-Giemsa stain, 40x

**Figure 6:**
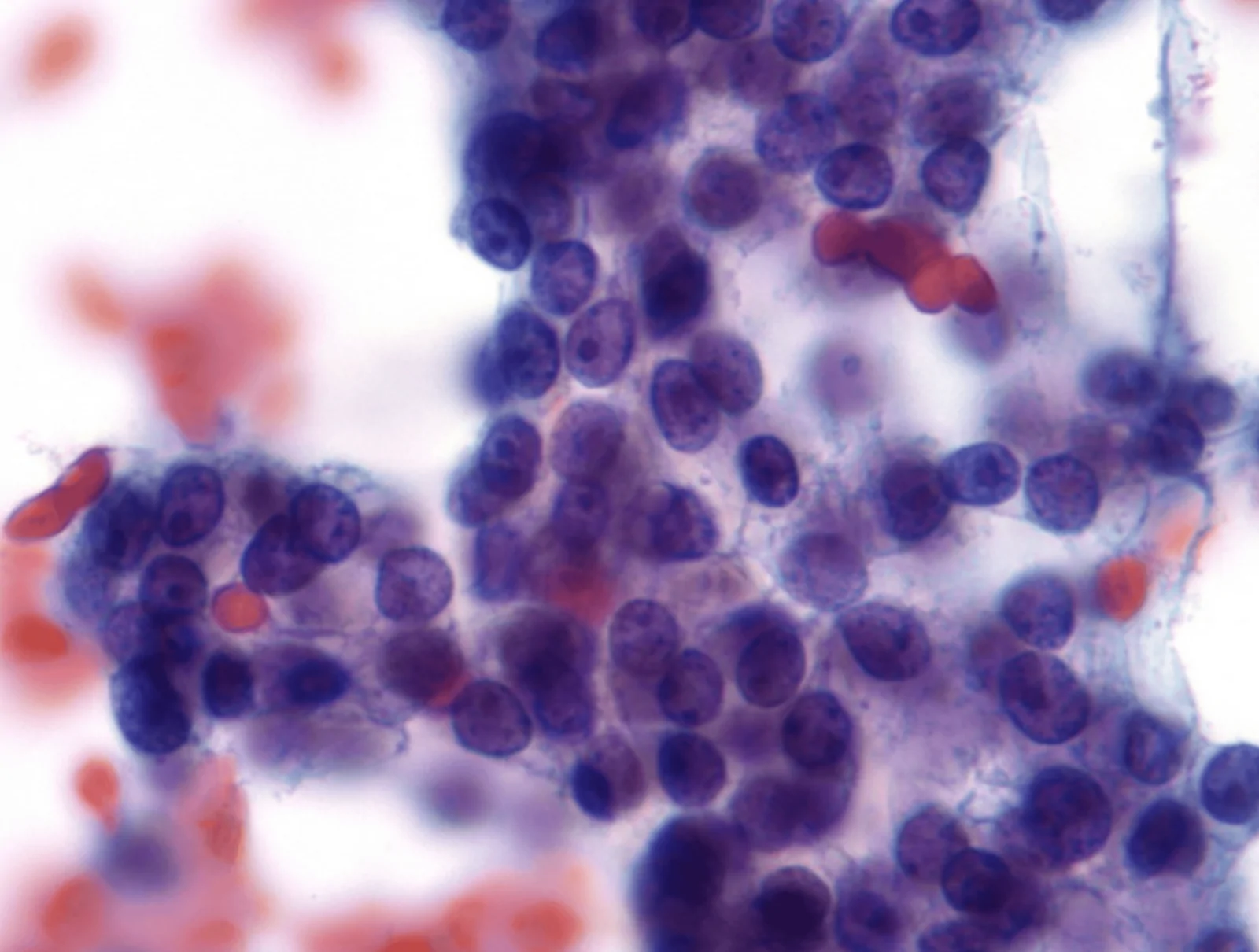
Crowding seen in follicular neoplasm May-Grünwald-Giemsa stain, 40x

**Figure 7:**
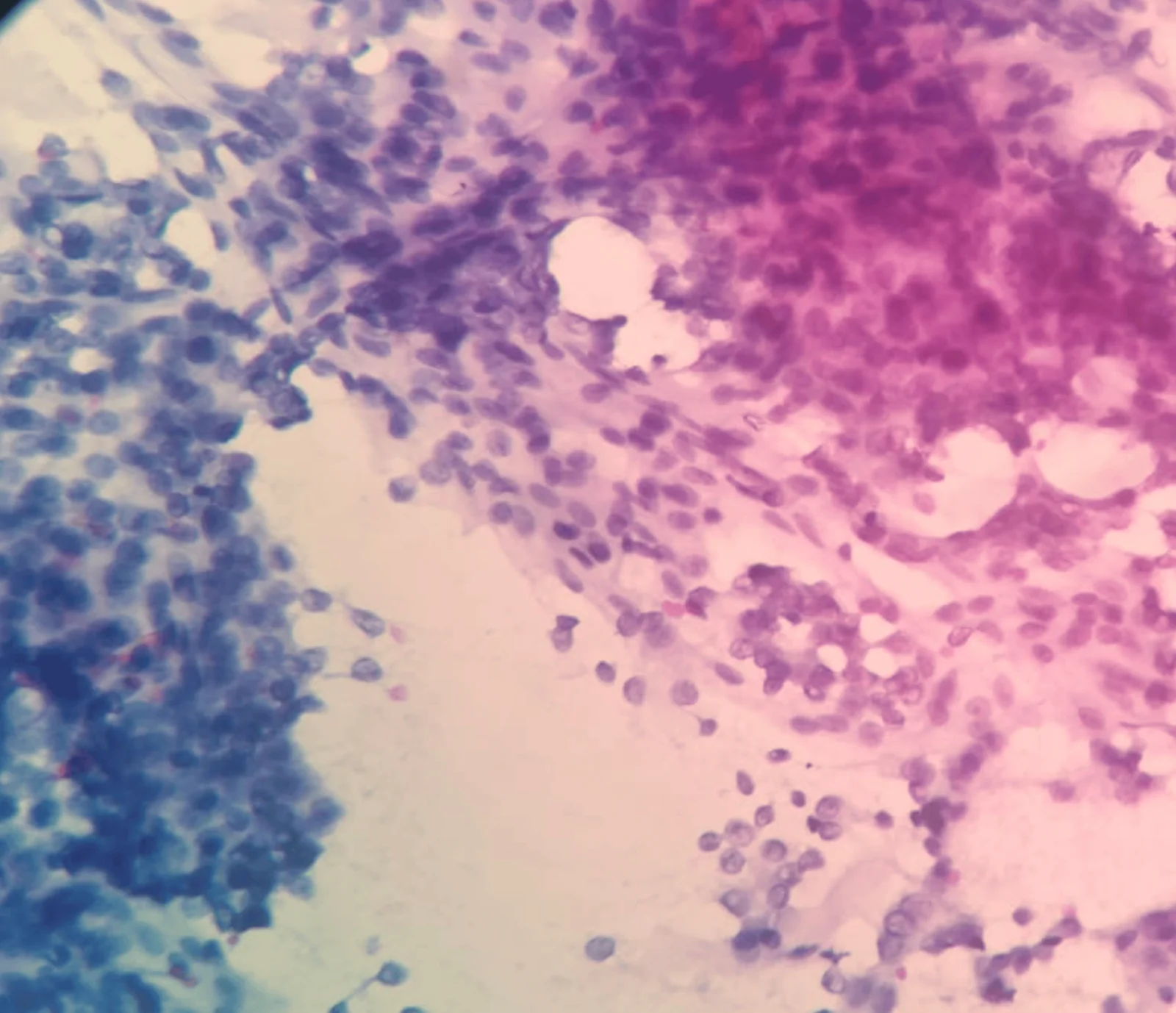
Suspicious of papillary carcinoma of thyroid May-Grünwald-Giemsa stain, 10x

**Figure 8:**
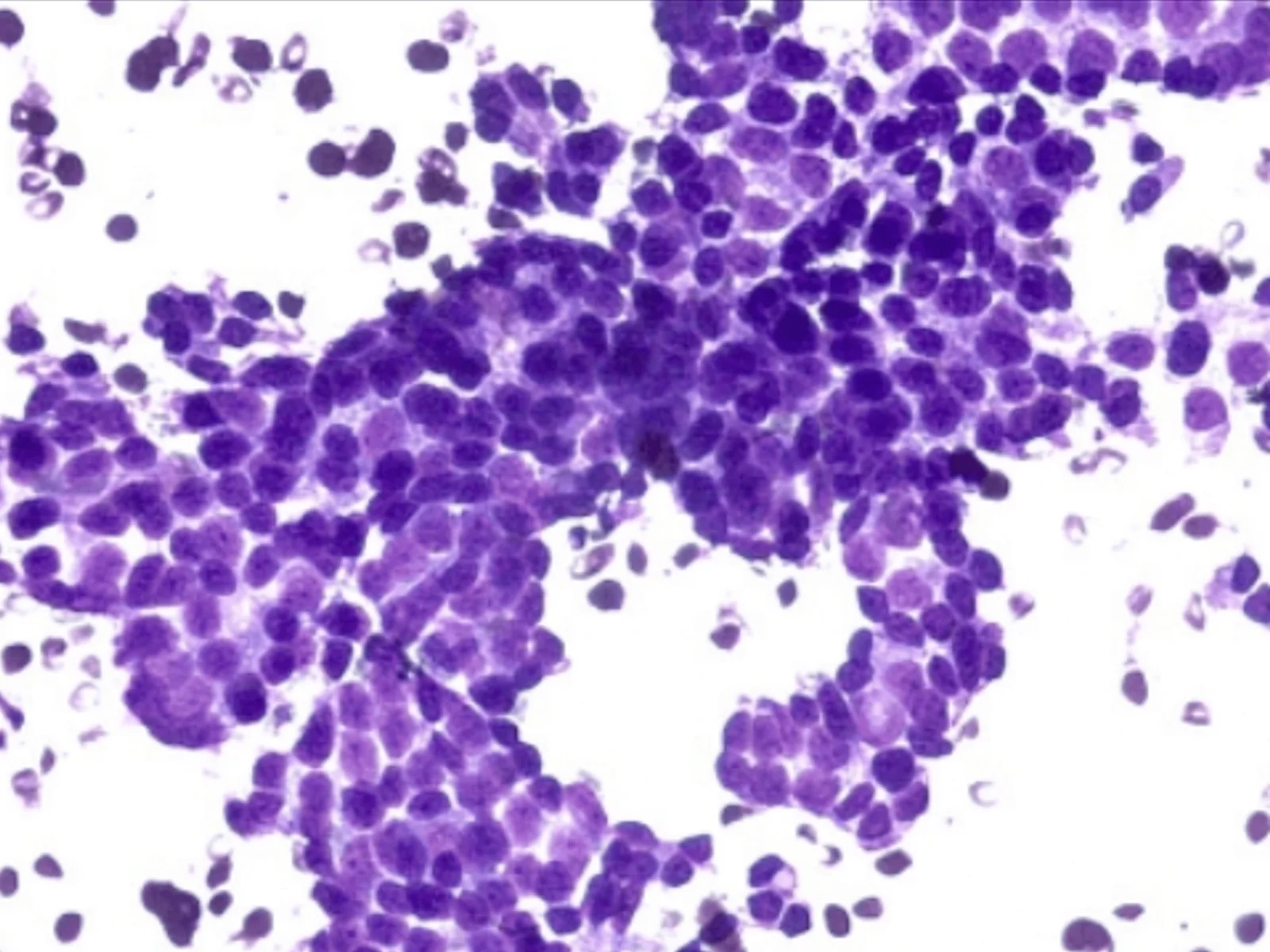
Suspicious of papillary carcinoma of thyroid May-Grünwald-Giemsa stain, 40x

**Figure 9:**
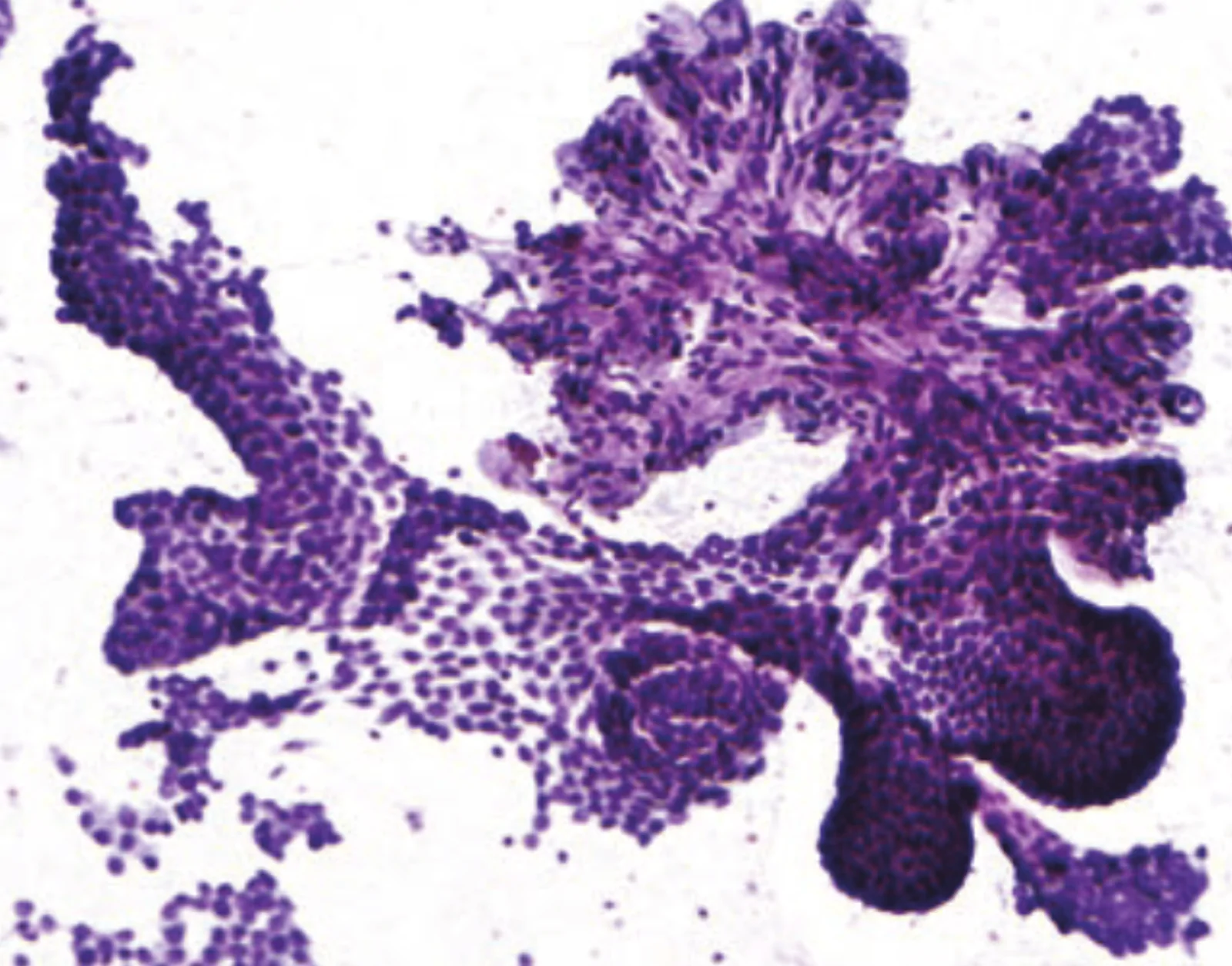
Papillary thyroid carcinoma- mix of flat sheets and rounded, papillary-like fragments with fibrovascular cores May-Grünwald-Giemsa stain, 10x
